# Visual Mismatch Negativity Reflects Enhanced Response to the Deviant: Evidence From Event-Related Potentials and Electroencephalogram Time-Frequency Analysis

**DOI:** 10.3389/fnhum.2022.800855

**Published:** 2022-03-08

**Authors:** Xianqing Zeng, Luyan Ji, Yanxiu Liu, Yue Zhang, Shimin Fu

**Affiliations:** Department of Psychology, Center for Brain and Cognitive Sciences, School of Education, Guangzhou University, Guangzhou, China

**Keywords:** emotion-related visual mismatch negativity, visual refractoriness, low-level features, memory trace hypothesis, visual mismatch oscillatory responses

## Abstract

Automatic detection of information changes in the visual environment is crucial for individual survival. Researchers use the oddball paradigm to study the brain’s response to frequently presented (standard) stimuli and occasionally presented (deviant) stimuli. The component that can be observed in the difference wave is called visual mismatch negativity (vMMN), which is obtained by subtracting event-related potentials (ERPs) evoked by the deviant from ERPs evoked by the standard. There are three hypotheses to explain the vMMN. The sensory fatigue (or refractoriness) hypothesis considers that weakened neural activity caused by repetition results in decreased ERPs of the standard. The memory trace hypothesis proposes that vMMN results from increased responses to the deviant. The predictive coding hypothesis attributes the difference to enhanced responses for deviants and suppression for standards. However, when distinguishing between these effects, previous researchers did not consider the effect of low-level features on the vMMN. In this experiment, we used face sequences composed of different emotions (e.g., neutral and fearful face) and presented an oddball sequence, a reverse oddball sequence, and an equiprobable sequence to participants. The deviant of the oddball sequence was subtracted from the standard of the oddball sequence, the reverse oddball sequence, and the same type of stimulus of the equiprobable sequence to get oddball-vMMN (vMMN1), reverse oddball-vMMN (vMMN2), and equiprobable-vMMN (vMMN3), respectively. The results showed no significant difference between vMMN2 and vMMN3 in 100–350 ms following stimulus onset, while the vMMN effect was significant, indicating that the probability of the standard did not affect vMMN, which supported the memory trace hypothesis. Additionally, the fearful-related vMMN were more negative than the neutral-related vMMN within the range of 100–150 ms, suggesting a negative bias. We analyzed the source location of different vMMNs. There was no significant difference in brain regions between different vMMNs. Time-frequency analysis showed that the deviant had stronger theta-band oscillatory than the standard (visual mismatch oscillatory responses, vMORs). However, there was no difference between vMORs2 and vMORs3, indicating that vMORs reflect an enhanced response to the deviant in terms of neural oscillation, supporting the memory trace hypothesis.

## Introduction

One of the important functions of the human cognitive system is to detect changes in the environment and make a response. Evidence that the brain can rapidly process discrete and unusual changes in events has been widely confirmed by studies using the event-related potentials (ERPs) technique (Kenemans et al., 2003). One crucial finding is that mismatch negativity (MMN), an ERP component, emerges due to violation of the probability in a sequence of sensory stimuli, reflecting the brain’s automatic detection of information changes (Kimura et al., 2011; Czigler, 2014). In early research, MMN was usually studied in the auditory domain (aMMN, Näätänen, 1990; Näätänen et al., 2001, 2005). However, many subsequent studies (Kimura, 2012; Stefanics et al., 2014b) have shown that the phenomenon also exists in the visual system, where it is known as visual mismatch negativity (vMMN). The vMMN is an ERP index of automatic processing of unattended visual information, which has been widely used to study the neural mechanism underlying a visual change detection process (Kimura et al., 2011; Stefanics et al., 2011; Li et al., 2012; Czigler, 2014; Kremláèek et al., 2016; Chen et al., 2020).

In vMMN studies, the passive oddball paradigm is often used to present events with high or low probability. In a sequence of visual stimuli (e.g., OOOXOOXOOO…), the frequently presented visual stimulus (e.g., O) is called the standard stimulus and the infrequent stimulus (e.g., X) is the deviant stimulus. In a reverse oddball sequence, the roles of the stimuli are reversed, i.e., the deviant becomes the standard, and the standard becomes the deviant. Comparison of the visual stimulus-evoked ERPs reveals more negative-going waveforms induced by deviant stimuli than those by standard stimuli at the posterior scalp electrodes from 150 to 400 ms following the stimulus onset (Kimura, 2012). The difference of the ERPs between the deviant and the standard stimuli is called vMMN. However, because of the differences in the presentation probability of deviant-standard stimulus pairs (D-S pairs), standard stimuli with high probability are more likely to cause refractoriness, such as S in SSSSD. It may also be D in DDDS. S is more likely to appear; S, therefore, establishes more repetitions. The difference wave may be affected by stimulus-specific refractoriness (Kimura et al., 2010; Stefanics et al., 2014b; Ruusuvirta, 2021).

Three hypotheses have been developed to explain vMMN: the sensory fatigue (or refractoriness) hypothesis, the memory trace hypothesis, and the predictive coding hypothesis. The sensory fatigue hypothesis holds that when stimuli are repeated, neural activity is usually weakened (Näätänen and Picton, 1987; Grill-Spector et al., 2006; Ruusuvirta, 2021). This repetition effect on neural activity has been confirmed using many methods, including the recording of individual cortical neurons (Caggiano et al., 2013; Kilner et al., 2014), the use of functional magnetic resonance imaging (Heleven and Overwalle, 2019; Rostalski et al., 2019), and the use of EEG/ERPs (Jonas et al., 2014; Stefanics et al., 2020). As mentioned above, when using the oddball paradigm to study the automatic processing of visual changes, the standard stimulus is repeatedly presented. The earliest visual refractoriness can occur 200 ms after the onset of the stimulus (Shen et al., 2017; Dravida et al., 2018; Feuerriegel et al., 2019). According to the sensory fatigue hypothesis, the vMMN effect is caused by decreased ERPs of the standard stimuli, not by increased responses to the deviant one (Nelken and Ulanovsky, 2007; May and Tiitinen, 2009).

The second hypothesis, known as the memory trace, holds that the vMMN is the result of automatic comparison between current and previous stimuli (Näätänen and Picton, 1987). The theory is that repetition of the standard stimuli would leave memory traces (or templates), and the brain would automatically compare the neural representation of the subsequent stimuli and discern the differences (Näätänen, 1990). While the brain would not respond to standard stimuli that match the traces, it would respond to a mismatch between deviant stimuli and the memory traces, resulting in deviant-related mismatch negativity. Therefore, from the perspective of memory traces, the vMMN effect is caused by the enhancement of the ERPs elicited by the deviant stimuli rather than the weakening ERPs elicited by the standard ones (Winkler, 2007; Paavilainen, 2013).

The memory trace hypothesis was interpreted as a “regularity violation” hypothesis by later researchers (Winkler, 2007). That is, the vMMN signal shows the difference between the current stimuli and the expectation based on previous information. It not only represents the memory traces but also the relationship between the previous stimuli (Stefanics et al., 2009, 2011; Paavilainen, 2013). Building on this, Garrido et al. (2008) posited a hierarchical predictive coding framework to explain the mismatch process. The predictive coding hypothesis assumes that the human brain is a nested system of different levels in which error signals are passed up and predictions are passed down. Repeated or interactive information transferred between different levels enables the model to be optimized or adjusted to select the optimal interpretation for the current sensory information input (Friston, 2005, 2010). According to the predictive coding hypothesis, the vMMN effect is caused by both the enhancement of the prediction error to the deviant stimuli and the weakening of the prediction error to the standard stimuli (Friston, 2005; Stefanics et al., 2014a,^2018^).

In the present study, we used the concept of “probability” rather than “violation of regularity” because “probability” is the description of the whole sequence, while “violation of regularity” is the microscopic description. The fact that stimuli with low probability violate the regularity established by stimuli with high probability is more likely to occur.

Some researchers have used the equiprobable paradigm to study vMMN and to control the refractoriness on vMMN (Czigler et al., 2002; Kimura et al., 2009, 2011; Kimura, 2012; Li et al., 2012; Kovarski et al., 2017). There are two kinds of sequences in the equiprobable paradigm, one oddball and one equiprobable. The oddball sequence is similar to the one in the oddball paradigm, including the deviant (with a probability of 20% occurrence, for example) and the standard stimuli (e.g., 80%). In the equiprobable sequence, the same deviant and standard stimuli as in the oddball sequence are used, and some other different stimuli are randomly intermixed, each with an equal probability (e.g., 20%), matching the probability of the deviant in the oddball sequences. The difference wave is obtained by subtracting the ERPs elicited by the deviant stimuli in the equiprobable sequence from the ERPs elicited by the same deviant stimuli in the oddball sequence, and these two ERPs are evoked by the same physical stimuli with the same probability. There is supposedly no regularity in the equiprobable sequence. Regularity in the sequence is the only difference between the deviant in the oddball sequence and control in the equiprobable sequence. Thus, the equiprobable vMMN is considered a genuine vMMN without stimulus-specific refractoriness effects (Stefanics et al., 2014b; Ding et al., 2020).

Previous studies of vMMN have used simple visual stimuli to study the effect of visual refractoriness on differential waves (Czigler et al., 2002; Kimura et al., 2009). Other studies used complex visual information (such as facial expression) as experimental materials, but the conclusion has been controversial (Li et al., 2012; Kreegipuu et al., 2013; Kovarski et al., 2017). For example, Li et al. (2012) and Kovarski et al. (2017) used the equiprobable paradigm to study the emotion-related vMMN effect. However, the results were partly inconsistent. Both studies presented equiprobable sequences and oddball sequences to the subjects. In the equiprobable sequence, faces with varied emotions were presented with equal probability, while in the oddball sequence, neutral faces (frequently presented) and fearful faces (or angry faces, infrequently presented) were presented. The ERPs elicited by the standard stimuli in the oddball sequence and the control stimuli in the equiprobable sequence were each subtracted from the ERPs elicited by the deviant stimuli in the oddball sequence, resulting in the oddball vMMN and control vMMN, respectively. Li et al. (2012) found that the early oddball vMMN (110–210 ms from stimulus onset) was more negative than the control vMMN suggesting that the oddball vMMN in the early stage was based on visual refractoriness, while the late oddball vMMN (210–310 ms) was not significantly different from the control vMMN suggesting that the oddball vMMN in the late stage was without refractoriness effects. However, Kovarski et al. (2017) found that the vMMN effect had no significant difference in both early and late stages. The deviant-standard stimulus pairs of the oddball vMMN showed differences in both presentation probability and low-level features, while the deviant-control stimulus pairs of the control vMMN had no differences in both dimensions. That is, it is unclear whether the difference between oddball vMMN and control vMMN is caused by the presentation probability (similar to refractoriness) or by the difference in low-level features. Therefore, further study is needed to explore this issue.

Our study adopted a variant of the oddball paradigm to explore the effects of visual refractoriness and low-level features on the vMMN effect. Specifically, participants were presented with an oddball sequence, a reverse oddball sequence, and an equiprobable sequence. The ERPs elicited by the standard stimuli in the oddball sequence, the standard stimuli in the reverse oddball sequence, and the deviant/control stimuli in the equiprobable sequence were each subtracted from the ERPs elicited by the deviant in the oddball sequence to obtain oddball vMMN (labeled vMMN1), reverse oddball vMMN (labeled vMMN2), and equiprobable, or control vMMN (labeled vMMN3), respectively. Previous studies used only two of these sequences, so the effect of visual refractoriness or low-level features could only be excluded. In the equiprobable paradigm, there were differences in low-level features and probability between the deviant and the standard stimuli in the oddball sequence (vMMN1), while in the equiprobable sequence, the pair had neither probability nor low-level feature differences (vMMN3). However, in the reverse oddball paradigm, the pair in vMMN2 had only probability difference. vMMN1 and vMMN3 were compared to verify whether visual refractoriness affected vMMN. In contrast, the deviant-standard stimulus pairs in vMMN1 and vMMN3 were different not only in the presentation probability (visual refractoriness) but also in low-level features. The pairs in vMMN2 and vMMN3 were different only in the presentation probability (i.e., visual refractoriness) but the same in low-level features, which allows a direct comparison of the true effects of visual refractoriness excluding low-level features.

Based on the memory trace hypothesis, we aimed to show that vMMN is not affected by ERP weakening of standard stimuli (visual refractoriness), so that there would be no significant difference between vMMN2 and vMMN3. Otherwise, based on the view of refractoriness (sensory fatigue hypothesis), the difference between vMMN2 and vMMN3 would be significantly different. In addition, our study focused on whether low-level features affect the vMMN effect, that is, whether there is a significant difference between vMMN1 and vMMN2.

Additionally, we investigated the effects of low-level features and refractoriness in the neural sources of vMMN. It has been suggested that the sources of vMMN are localized in the superior frontal gyrus, middle frontal gyrus, middle occipital gyrus, inferior parietal gyrus, medial temporal lobe, superior temporal gyrus, cingulate gyrus, insula, and precuneus (Li et al., 2012; Xu et al., 2013; Vogel et al., 2015; Chen et al., 2020). We anticipated that differences in low-level features and refractoriness would affect the sources in these regions of the brain. We also explored the visual mismatch neural oscillatory responses (vMORs). Presently, few studies have focused on vMORs. Task-irrelevant deviant stimuli induce greater alpha oscillation (Tugin et al., 2016) or greater theta oscillation (Hesse et al., 2017; Yan et al., 2017) compared to the standard stimuli. The evidence so far is mixed (Stothart and Kazanina, 2013; Hesse et al., 2017; Yan et al., 2017; Chen et al., 2020). Most studies focus on the power of vMORs in the alpha band (8–13 Hz) and theta band (4–7 Hz). Therefore, our study focused on the oscillatory response of vMMN in both alpha and theta bands.

## Materials and Methods

### Participants

Twenty-one students were recruited from Guangzhou University and participated for payment or course credit. One participant’s data were not used because the impedance was difficult to drop below 5,000 Ω. The final sample comprised 20 participants (10 males, 10 females, mean age = 19.30 years, *SD* = 1.20 years). All participants were right-handed, with normal or corrected-to-normal vision. They were naive about the purpose of the experiment and all participants signed informed consent before the experiment. After the experiment, we told them that the sequence of faces was made up of different expressions. The study was approved by the local institutional research board (IRB) of the Department of Psychology, School of Education, Guangzhou University.

### Materials and Procedure

The stimuli consisted of 10 human faces of different identities (5 males, 5 females). The emotions include fearful, anger, happy, surprise, and neutral. All face pictures were selected from the NimStim Database (Tottenham et al., 2009). According to NimStim, fearful face are correctly identified by 72% of observers. Other rates of correct identification are listed as 87% for angry faces, 98% for happy faces, 82% for surprise faces, and 57% for neutral faces. Each gray picture extended to a visual angle of approximately 6°horizontally and 6°vertically.

**FIGURE 1:**
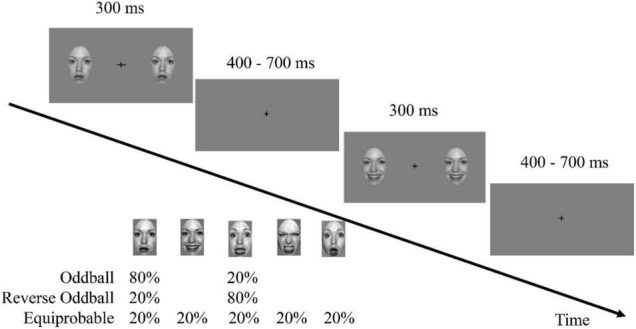
A task schematic of the sequence shows the time course of the stimulus presented for 300 ms, followed by the cross displayed on a gray screen for 400–700 ms.

Participants sat comfortably in a dimly lit and electrically shielded room, at a viewing distance of approximately 60 cm. The experiment was implemented using E-Prime software (version 3.0, Psychology Software Tools, Inc., Pittsburgh, United States). The stimuli were presented on a 17-inch cathode ray tube monitor with a refresh rate of 85 Hz and a resolution of 1,024 × 768 pixels. At the beginning of each trial, two faces identical in features and emotions with dimensions of 0.5° × 0.5°were presented to the left and the right of the fixation cross. A distance of 6°separated the center of the face picture and the fixation cross. In each trial, the face image disappeared after 300 ms and then the screen presented only the fixation cross for 400–700 ms (Figure 1). As in previous vMMN studies (Stefanics et al., 2012; Chen et al., 2020), participants were asked to focus on the fixation cross during the display of faces and to discriminate and respond to changes in the fixation cross. The frequency of change of the fixation cross was 0.15. When it changed, the horizontal or vertical line could grow from 0.5 to 1.0° for 300 ms.

The experimental procedure consisted of three sequences. The oddball sequence comprised 576 trials of neutral faces (standard stimulus, presented at 80% probability) and 144 trials of fearful faces (deviant stimulus, 20%). The reverse oddball sequence comprised 576 trials presenting fearful faces and 144 trials of neutral faces. The equiprobable sequence comprised 144 trials (20%) each for fearful, angry, happy, surprised, and neutral faces. To exclude the influence of differences in low-level features from the influence of emotions, the faces in each trial were randomly presented, and the order of the trials also was randomly determined. At least one standard stimulus was presented between two deviant stimuli. The participants were asked not to blink while viewing the sequence in the experiment. To prevent fatigue, each sequence was divided equally into 6 blocks, each lasting about 1.5 min, during which the participants could determine the duration of the rest time. The presentation order of the three sequences among the participants was balanced.

### Recordings

Electroencephalogram (EEG) recordings were done with Ag/AgCl electrodes placed at 64 locations on an elastic electrode cap (NeuroScan, Texas, United States). The nose tip served as the online reference electrode. The horizontal electrooculogram was recorded by placing two electrodes 1 cm from the external canthi of the left and right eye. The vertical electrooculogram was recorded by placing two electrodes 2 cm above and below the left eye. The EEG data were sampled at a rate of 1,000 Hz and were filtered online (0.05–100 Hz bandpass). The impedance of all electrodes was maintained at a level below 5,000 Ω throughout the recording session.

The EEG data were bandpass filtered offline at 0.1–30 Hz with a Hamming window finite impulse response filter (24 dB/oct slope). Epochs from −200 to 699 ms after stimulus onset were extracted from the continuous EEG data. Data in the interval from −200 ms to stimulus onset served as the baseline. Independent component analysis was used to identify artifact components related to eye movements. The following epochs were excluded: (1) epochs that contained changes in the fixation cross, (2) epochs that contained participants’ responses, (3) epochs with potential values exceeding ± 75 μV on an EEG channel. The frequency of rejected epochs was 28.4% (*SD* = 5.8%). There were 299–470 effective standard trials and 65–115 effective deviant trials remaining.

## Analysis

### Behavior Data

To test whether the participants were in a state of inattention to faces, this study analyzed the response accuracy in behavioral data. If the subject paid attention to the fixation cross, high percentage changes in the fixation cross gaze should be captured, otherwise, the hit rate would be low or the false alarm rate would be high. We did not analyze response time because we were most interested in accuracy rather than reaction time.

### Event-Related Potentials and Difference Waves

The EEG signals of six kinds of conditions, characterized by the combination of stimulus type (deviant and standard) and sequence type (oddball sequence, reverse oddball sequence, and equiprobable sequence), were superimposed and averaged, and the corresponding ERP waveforms were obtained within the time interval of −200 through 699 ms.

In vMMN studies, the difference wave is obtained by subtracting the ERP of the standard stimulus (or the ERP of the corresponding face in the equiprobable sequence) from the ERP of the deviant stimulus. Previous studies have shown that the differential wave generally peaks in the parietal occipital region within 100–400 ms (Kimura et al., 2011; Kimura, 2012). Three kinds of vMMN were calculated by subtracting the ERP of the standard (or control) stimulus in different sequences from that of the deviant stimulus in the oddball sequence. Here we use the shorthand Fearful 1, Fearful 2, and Fearful 3 and Neutral 1, Neutral 2, and Neutral 3 in describing the values for different emotion types. Specifically, Neutral 1 (80%, the standard) in the oddball sequence was subtracted from Fearful 1 to obtain the fearful face vMMN1. To obtain vMMN2, Fearful 2 (80%, the standard) was subtracted from Fearful 1. For vMMN3, Fearful 3 (20%, the equiprobable stimuli) was subtracted from Fearful 1. According to the MMN calculation method, the neutral face in the reverse oddball sequence (Neutral 2) can also be used as the deviant stimulus. Therefore, neutral face vMMN1, neutral face vMMN2, and neutral face vMMN3 were obtained by subtracting the amplitude for Neutral 2 from Fearful 2, Neutral 1, and Neutral 3, respectively.

In the subsequent discussion in this manuscript, the six vMMNs are abbreviated as FvMMN1, FvMMN2, FvMMN3 for fearful face-related vMMNs, and NvMMN1, NvMMN2, and NvMMN3 for neutral faces.

Based on the data from previous vMMN studies and the present study, we selected as the analysis object the mean ERP amplitude within the 101–350 ms range in the parietal-occipital region (labeled PO3, PO5, PO7 in the left hemisphere and PO4, PO6, PO8 in the right hemisphere). We used repeated-measure analysis of variance (ANOVA) to examine the main effects and interactions of different factors. Factors we considered were stimulus type (deviant, standard), emotion type (fearful face, neutral face), sequence of standard stimulus (vMMN1, vMMN2, vMMN3), and hemisphere (left hemisphere, right hemisphere). According to previous studies, vMMN generally appears in the range of 100–350 ms. In this study, a flexible time processing method with 5 50-ms-long time intervals was adopted to measure the mean amplitude of ERP in the range of 101–350 ms, and the time interval was taken as a factor in the ANOVA. Therefore, a five-factor repeated-measure ANOVA was performed for the ERP amplitude, which included stimulus type × emotion type × sequence × hemisphere × time interval. The *p*-value was corrected using the Greenhouse-Geisser method when necessary, but the degree of freedom was reported as the uncorrected value. LSD correction was used for *post hoc* comparisons. Time-frequency analysis uses the same method. Since the object of this study was the mismatched negative wave, we considered only the main effect or interaction of the stimulus type.

### Source Localization Analysis

Source localization can identify and map brain regions associated with specific stimulus responses or behaviors, further improving spatial resolution from the analysis of sensor patterns (Baillet et al., 2001). In this study, we used standardized low-resolution electromagnetic brain tomography to analyze the source locations of three kinds of vMMN with different emotions (Pascual-Marqui, 2002). Using the ICBM152 template to compute the head model (Fonov et al., 2009), we calculated the cortical three-dimensional distribution of the scalp EEG current source density by constrained solution. This method provides a standardized, discrete, three-dimensional, distributed, linear, minimum-norm inverse solution to the inverse problem of the brain source location. However, it locates only the gray matter of the brain and does not take into account the depth of the source.

The source analysis started from the trial level. The noise covariance matrix was calculated within the range of −200 to −1 ms, and the source activity from 0 to 699 ms was calculated using a unified head model. Data for the source activity of each subject under each condition were obtained by stacking the average of each trial. Data for source activity in the time range −200 to −1 ms were taken as the baseline, and the source activity data of 0–699 ms were converted into a *Z*-value, then spatial smoothing was performed with a resolution of 3 mm. To examine the source locations of different vMMNs, the source in the given time interval would be averaged to a normalized value (Z-score transformation, the source activity of −200 to −1 ms would be served as baseline). The paired permutation *t*-test was carried out between the normalized map induced by the deviant and the normalized map induced by the standard. To test the difference of source locations of vMMN with different emotions and different sequences, the difference between the source induced by the deviant and the standard was calculated, using the formula *Source*_*vMMN*_=*Z*_*D*−*s*_. Paired permutation *t*-test was used to compare the sources of vMMN1, vMMN2, and vMMN3 with different emotions.

The statistical significance test was conducted using the Monte-Carlo approach in the paired permutation tests, using 5,000 random samples. The results of multiple comparisons between source signals were corrected for false discovery rate, and the critical threshold of a two-tailed test with an alpha level of 0.05 was used to determine whether the difference was significant.

### Time-Frequency Analysis

The Morlet wavelet transform method was used for time-frequency decomposition (Bertrand and Tallon-Baudry, 2000). First, to obtain the time-frequency average of each trial, we calculated the non-standardized time-frequency power within the time range -200 to 699 ms of the trial level, using the time resolution of 2 s and the center frequency of 0.5 Hz, and averaged the result. To keep the time-frequency signal stable, the average value of all tests was calculated, and the average value of each test was subtracted before calculating the time-frequency power. Then, using the range of -200 to 0 ms as the baseline, we used decibel conversion to normalize the time-frequency power data within the 0–699-ms range. The conversion formula is *dB* = 10 × lg (energy after baseline / average energy baseline), and we obtained the time-frequency energy graphs of the standard and the deviant stimuli. Then, we calculated the difference between the deviant (D) and the standard (S) values to obtain the visual mismatch oscillatory responses (vMORs), using the formula *vMORs* = *dB*_*D*_−*dB*_*S*_.

Similar to the ERP analysis, our study focused on the dB values of the theta band (4–7 Hz) and alpha band (8–13 Hz) in the parietal-occipital region, labeled PO3, PO5, and PO7 in the left hemisphere and PO4, PO6, and PO8 in the right (Chen et al., 2020). Data from different electrode points in the same hemisphere were averaged point-to-point. The main effects and interactions of different factors were examined by repeated-measure ANOVA, with the factors of the frequency band (theta, alpha), emotion type (fearful, neutral), sequence (vMORs1, vMORs2, and vMORs3), hemisphere (left, right), time interval (7–100-ms-long consecutive intervals in the 0–699-ms range) and the type of stimulus (deviant, standard). We aimed to study the vMORs, therefore we focused only on the main effect of the stimulus type or its interaction.

Repeated-measure ANOVA was performed using SPSS 23.0 software. EEGLAB (Delorme and Makeig, 2004) and ERPLAB (Calderon and Luck, 2014) were used to preprocess EEG and extract ERP values. Source location analysis, time-frequency analysis, and permutation tests were performed with the Brainstorm3 toolbox (Tadel et al., 2011). The EEGLAB, ERPLAB, and Brainstorm3 functions were accessed in MATLAB 2016b (MathWorks).

## Results

### Behavioral Data

The behavioral data in the experiment showed that the average correct rate of fixation change detection was 97% (85–99%, *SD* = 2.8%), suggesting that participants focused on the fixation cross, leaving task-irrelevant emotional faces unattended.

### Event-Related Potentials and Difference Waves

**FIGURE 2:**
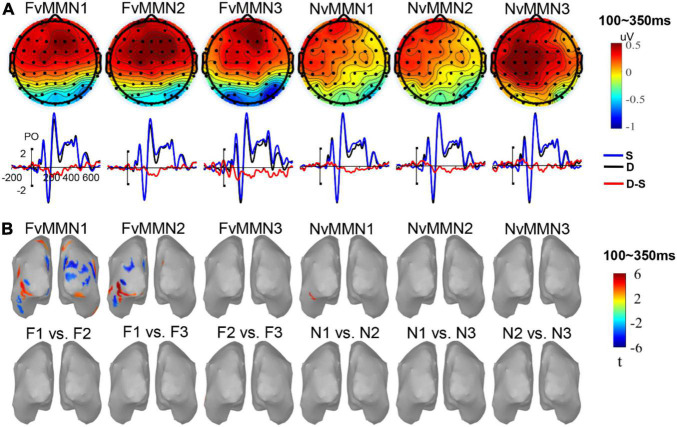
**(A)** The vMMN scalp maps and waveforms in parieto-occipital areas. **(B)** The significant source activation of vMMN and significant source activation of paired permutation *t*-test of different vMMN (no significant region was found).

Figure 2A shows the ERP waveforms of vMMN1, vMMN2, and vMMN3 for fearful and neutral emotion types in the parietal-occipital region, and the topographic map of the average amplitude within the 100–350-ms range. Consistent with previous studies on vMMN, the difference wave emerged within that time range.

The ANOVA revealed that the main effect of stimulus type was significant $F\,\left(1,19\right)=4.80,p=0.041,\eta_{p}^{2}=0.202$. The ERP amplitude induced by the deviant was significantly larger than that of the standard, indicating a significant vMMN in the parietal-occipital region during the time range 101–350 ms. The interaction between stimulus type and time interval was significant, $F\,\left(4,76\right)=2.76,p=0.047,\eta_{p}^{2}=0.127.$ *Post hoc* analysis found that the interaction was mainly caused by vMMN within 251–300 ms [$F\,\left(1,19\right)=8.18,p=0.010,\eta_{p}^{2}=0.301$], 201–250 ms [$F\,\left(1,19\right)=4.75,p=0.042,\eta_{p}^{2}=0.200$], and 301–350 ms [$F\,\left(1,19\right)=4.57,p=0.046,\eta_{p}^{2}=0.194$], and 0.0.164 *ps* = 0.634 in other time intervals. The interaction of stimulus type × hemisphere was significant, $F\,\left(1,19\right)=8.11,p=0.010,\eta_{p}^{2}=0.299$. *Post hoc* analysis showed that the interaction was mainly caused by the vMMN effect of the right hemisphere [$F\,\left(1,19\right)=6.75,p=0.018,\eta_{p}^{2}=0.262$], indicating that vMMN had right hemisphere dominance.

**FIGURE 3:**
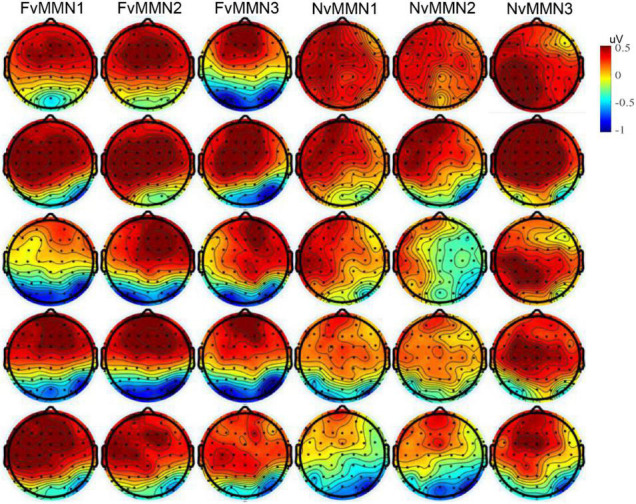
From top to bottom: The vMMN scalp maps of mean amplitude in the time range of 101–150, 151–200, 201–250, 251–300, and 301–350 ms.

In addition, the interaction of stimulus type × emotion type × time interval × hemisphere was marginally significant, $F\,\left(4,76\right)=2.66,p=0.057,\eta_{p}^{2}=0.123$, indicating that the hemisphere effect of emotional vMMN varied by time interval. Since the emotional effect of vMMN was one of our research issues, we conducted data analysis in five time windows, respectively, and conducted a three-factor repeated-measure ANOVA of stimulus type × emotion type × hemisphere (for scalp distribution, see Figure 3).

In the range of 101–150 ms, the interaction between stimulus type × emotion type was significant $\left[F\left(1,19\right)=5.54,p=0.030,\eta_{p}^{2}=0.226\right)]$, and the vMMN effect of fearful emotion $\left[F\,\left(1,19\right)=5.05,p=0.037,\eta_{p}^{2}=0.210\right]$ was significantly greater than that of neutral emotion (*p* = 0.302). From 151 to 200 ms, the interaction of stimulus type × hemisphere was significant $\left[F\,\left(1,19\right)=7.62,p=0.012,\eta_{p}^{2}=0.286\right]$. The vMMN effect of the right hemisphere $\left[F\,\left(1,19\right)=5.15,p=0.035,\eta_{p}^{2}=0.213\right]$ was significantly greater than that of the left hemisphere (*p* = 0.564). The vMMN effect was significant $\left[F\,\left(1,19\right)=4.75,p=0.042,\eta_{p}^{2}=0.200\right]$within 201–251 ms. The interaction of stimulus type × hemisphere was significant $\left[F\,\left(1,19\right)=4.77,p=0.042,\eta_{p}^{2}=0.201\right]$. The vMMN effect of the right hemisphere $\left[F\,\left(1,19\right)=6.24,p=0.022,\eta_{p}^{2}=0.247\right]$was significantly greater than that of the left hemisphere (*p* = 0.099). The interaction of stimulus type × emotion type × hemisphere was marginally significant $\left[F\,\left(1,19\right)=3.89,p=0.063,\eta_{p}^{2}=0.170\right]$, and the negative bias of the left hemisphere $\left[F\,\left(1,19\right)=4.18,p=0.055,\eta_{p}^{2}=0.180\right]$was significantly higher than that of the right hemisphere (*p* = 0.412). In the time range 251–300 ms, only the main effects of stimulus type were significant $\left[F\,\left(1,19\right)=8.18,p=0.010,\eta_{p}^{2}=0.301\right]$. From 301 to 350 ms, the vMMN effect was significant $\left[F\,\left(1,19\right)=4.57,p=0.046,\eta_{p}^{2}=0.194\right]$, stimulus type × hemispheric interaction was significant $\left[F\,\left(1,19\right)=7.47,p=0.013,\eta_{p}^{2}=0.282\right]$, and the vMMN effect of the right hemisphere $\left[F\,\left(1,19\right)=6.60,p=0.019,\eta_{p}^{2}=0.258\right]$ was greater than that of the left hemisphere (*p* = 0.141).

A *post-hoc* test of four-factor repeated-measure ANOVA mentioned above found negative bias and right hemisphere dominance of the vMMN effect, which is typical in emotional vMMN studies. The remaining five-factor repeated-measure ANOVA showed no significant interaction with the type of stimulus (0.102 < ps < 0.713).

### Source Localization Analysis

Source location and source location differences (Figure 2B) were tested for average vMMN activity within 100–350 ms. Sources of FvMMN1 were located in the lateral occipital lobe, inferior parietal lobe, superior parietal lobe, inferior temporal lobe, cuneus, and precuneus on both sides of the brain. Sources of FvMMN2 were located in the lateral occipital lobe, inferior temporal lobe, inferior parietal lobe, fusiform gyrus, glossal gyrus, precuneus, and right cuneus. The source of NvMMN1 was located in the left fusiform lobe. FvMMN3, NvMMN2, and NvMMN3 had no significant activation regions.

An examination of source location differences revealed that no differences were found in vMMN1 vs. vMMN2 nor vMMN1 vs. vMMN3 nor vMMN2 vs. vMMN3, regardless of emotion (Figure 2B), indicating that, according to the average activity in the range of 100–350 ms, the low-level features and repetition effect of the standard (or visual refractoriness) did not affect the source of difference of vMMN.

The paired permutation *t*-test showed no significant differences in FvMMN1 vs. NvMMN1, FvMMN2 vs. NvMMN2, and FvMMN3 vs. NvMMN3 across the whole brain range, suggesting no significant differences in the sources of different emotion-related vMMN.

### Time-Frequency Analysis

**FIGURE 4:**
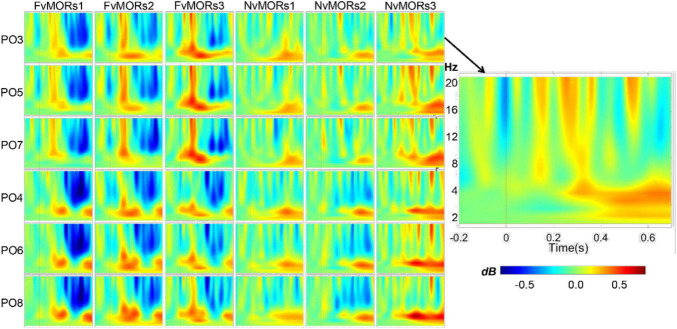
Visual mismatch oscillatory responses in different emotion and sequence conditions at electrode sites of parieto-occipital areas.

Figure 4 shows the time-frequency maps of different vMORs in the parietal-occipital region. Repeated-measure ANOVA for time-frequency data showed that the main effect of stimulus type was not significant [$F\,\left(1,19\right)=0.244,p=0.627,\eta_{p}^{2}=0.013$]. The interaction of stimulus type × frequency band was significant [$F\,\left(1,19\right)=12.30,p=0.002,\eta_{p}^{2}=0.393$]. *Post hoc* analysis showed that the vMORs of the theta frequency band (mean 0.148, 95%CI: [0.009 0.287], *p* = 0.038) were significantly larger than that in the alpha band (-0.091, 95%CI: [-0.231 0.048], *p* = 0.187). The interaction of stimulus type × frequency band × time interval was significant [$F\left(6,114\right)=2.71,p=0.048,\eta_{p}^{2}=0.125]$. *Post hoc* analysis showed that the vMORs in the theta band were positive (*ps* = 0.052) in the 100–199 and 200–299-ms ranges, while the vMORs in the alpha band were not significant (*ps* = 0.222). The vMORs of the alpha band were negative (*ps* = 0.015) and the theta band was not significant (*ps* = 0.193) in the 400–499 and 500–599-ms ranges. The interaction of stimulus type × emotion × time interval was significant [$F\,\left(6,114\right)=2.84,p=0.050,\eta_{p}^{2}=0.130$]. *Post hoc* analysis showed that within 100–199 ms, the vMORs of the fearful face were significantly positive [$F\left(1,19\right)=5.76,p=0.027,\eta_{p}^{2}=0.233]$, while those of neutral face were not (*p* = 0.860). The vMORs of the fearful face (*ps* = 0.074) and neutral face (*ps* = 0.307) in other time intervals were not significant. The interaction of the six factors was marginally significant $\left[F\,\left(12,228\right)=2.17,p=0.066,\eta_{p}^{2}=0.103\right]$, and the results were no longer analyzed.

## Discussion

The results showed significant vMMN in the time range of 100–350 ms and vMORs effects in the theta band, as well as a negative bias. However, there were no significant differences among the three kinds of responses measured by the oddball (vMMN1/vMORs1), the reverse oddball (vMMN2/vMORs2), and the equiprobable paradigms (vMMN3/vMORs3), regardless of emotion type. The results suggest that low-level physical information and the repetition effect (i.e., refractoriness) did not affect vMMN (vMORs). In addition, the source localization showed consistent results.

### Different Paradigms in the Visual Mismatch Negativity Study

We found that there was no significant difference in the effects of vMMN among different sequences, indicating that vMMN from all three paradigms can be used as an indicator of automatic detection.

Previous studies on automatic processing of visual information generally used the oddball paradigm (Zhao and Li, 2006; Astikainen and Hietanen, 2009), the reverse oddball paradigm (Stefanics et al., 2012; Chen et al., 2020), or the equiprobable paradigm (Czigler et al., 2002; Kimura et al., 2009; Li et al., 2012; Kovarski et al., 2017). In the oddball paradigm, there are differences in low-level features between the deviant and the standard stimuli, and the probability of these two kinds of stimuli also is different. With the reverse oddball paradigm, only the difference in probability is present. In the equiprobable paradigm, there are neither differences in probability nor differences in the low-level features. Our evidence showed no significant differences in the vMMN effects among the three paradigms. The differences of low-level features and the differences of probability in the deviant-standard stimulus pair seemed not to affect the vMMN effects, suggesting that the use of vMMN is an effective and robust index of automatic detection.

The current study obtained a significant vMMN effect, which was not affected by low-level physical features. This result was contrary to the conclusion of a recent study. Male et al. (2020) repeated the experiment (orientation vMMN) of Kimura et al. (2009) and studied the vMMN effects of contrast, phase, and spatial frequency. There was no significant deviant-minus-same standard vMMN effect, but a significant early ERP that might index low-level visual irregularities, i.e., N1 and P1, was found. A potential possibility is that, as suggested by Male et al. (2020), vMMN cannot reflect the automatic processing of low-level visual features. If so, facial emotion-related vMMN obtained in this study should not belong to this category. In fact, the latency of ERP induced by face are later, such as N170 and N250. The time window of these components is very consistent with that of vMMN effect. However, this conclusion needs more targeted research to confirm.

### Memory Trace Rather Than Refractoriness Accounts for Visual Mismatch Negativity

Our results suggest that the vMMN effects in the time range of 100–350 ms are not affected by the repetition effect, indicating that vMMN index is an enhanced response to the deviant.

This is consistent with the findings of Kovarski et al. (2017) but does not fully support the findings of Kimura et al. (2009) and Li et al. (2012), which concluded that the early (before 210 ms) vMMN is a repetition effect. These studies compared responses to oddball (vMMN1) and control (vMMN3) sequences in the same group of participants. However, the studies seemed to confuse the low-level information effect with the repetition effect, because the deviant-standard stimuli pairs were not different in the presentation probability (e.g., 20 and 80% in vMMN1, 20 and 20% in vMMN3) and also in the low-level information (e.g., anger face for deviant and neutral face for standard in vMMN1, and anger face for both deviant and standard in vMMN3). In the current study, the deviant stimulus in vMMN2 (vMORs2) and vMMN3 (vMORs3) was the fearful face in the oddball sequence (20%), while the standard stimulus was the fearful face in the reverse oddball sequence (80%) and the fearful face in the equiprobable sequence (20%), which differed only in probability. Therefore, the differences between vMMN2 (vMORs2) and vMMN3 (vMORs3) can be assumed to be the result of the repetition effect. According to the view of refractoriness (Nelken and Ulanovsky, 2007; May and Tiitinen, 2009), repetition of the standard stimulus weakens neural activity, resulting in the emergence of vMMN. However, the current study did not find that repetition of the standard stimulus had a significant influence on vMMN. Our results indicate that the vMMN effect is not due to refractoriness of repeated stimuli, but rather an enhanced response to rare stimuli, which supports the memory trace hypothesis (Winkler, 2007; Paavilainen, 2013). The results favor theoretical accounts that describe vMMN as an indicator of “memory trace” (Winkler, 2007; Paavilainen, 2013) rather than refractoriness accounts (Nelken and Ulanovsky, 2007; May and Tiitinen, 2009) or predictive coding view (Stefanics et al., 2014a,^2018^).

A recent study has also examined the effect of repetition on visual mismatch responses (VMRs, (Feuerriegel et al., 2021). Feuerriegel et al. (2021) used the fast visual stimulus presentation paradigm. In the sequence, the base face stimuli appeared periodically at a frequency of 6 Hz. Either the expected face (standard stimulus) or a surprise face (deviant stimulus) appeared every 7 faces. The probability of the surprise stimulus in each sequence varied from 10 to 90% and that of the expected stimulus varied from 90 to 10%. Therefore, there were 50% neutral stimuli (corresponding to the equiprobable stimuli). The results showed that in the parieto-occipital region, ERPs induced by the surprise stimuli were significantly more negative than those induced by the expected stimuli, meaning that VMRs were observed. On the other hand, there were no significant differences between the neutral (50%) and expected stimuli (90%), suggesting that VMRs reflect an enhanced response to the surprise stimuli rather than a decreased response to the expected stimuli. In this study, we compared ERPs of the deviant (20%) with those of the equiprobable (20%) and also showed a significant vMMN, which was consistent with Feuerriegel et al. (2021). To be noted, the control of visual refractoriness was stricter in the current study.

Several studies have shown that task-irrelevant deviant stimuli (e.g., line orientation or facial emotion) can lead to greater alpha oscillations (Tugin et al., 2016; Chen et al., 2020). Some researchers considered that the enhancement of the alpha band was the inhibition of response to stimuli (Klimesch, 2012). Some studies have also found that deviant stimuli induced larger theta-band oscillations (Stothart and Kazanina, 2013; Hesse et al., 2017; Yan et al., 2017). Our results were similar to Stothart and Kazanina (2013) in that a deviant emotional face induced greater theta oscillation and smaller alpha oscillation.

However, vMORs were not affected by the presentation probability of the standard stimuli, indicating that vMORs reflect increased oscillation with the deviant stimuli rather than reduced oscillation with the standard stimuli. Combined with the results of ERP, we found that the changes in visual information caused the enhancement of both amplitude and neural oscillation. Some researchers believe that theta-band oscillation is related to facilitatory and inhibitory attention (Karakas, 2020), or an inherent feature of the attention network in support of top-down guided attention (Helfrich et al., 2019). For the deviant stimuli, a stronger inhibition mechanism of the brain was required, compared to frequently presented standard stimuli.

In addition, the results of source analysis showed that the sources of different vMMN had no significant difference in the whole brain, which was consistent with the results of time-frequency analysis and ERP. In general, we got relatively consistent results that the repetition effect of standard stimuli did not affect vMMNs/vMORs, suggesting that vMMNs/vMORs index the deviant stimuli, supporting the memory trace view of vMMNs/vMORs.

### The Negative Bias of Emotion-Related Visual Mismatch Negativity

We found that the vMMN effect of fearful faces was significantly greater than that of neutral faces within 100–150 ms, indicating that the brain has a negative bias toward the automatic processing of emotions.

Negative emotion is related to a negative internal state, and the emergence of negative emotion may be related to the brain’s perception of dangerous information. Recently, studies have shown that relative to positive emotions, negative emotions may be processed automatically at a lower level of consciousness (Chen et al., 2020) and may more easily break through the threshold of unconsciousness (Jiang et al., 2018), and may more quickly affect the amygdala (Méndez-Bértolo et al., 2016). We found that among the emotion-related vMMN studies, three studies used fearful- or angry-neutral face stimuli pairs (Kovarski et al., 2017; Rosburg et al., 2019; Kask et al., 2021). Consistent with these studies, the fearful-related vMMN effect is significantly greater than the neutral-related one. In addition, we analyzed the neural oscillatory response and found that vMORs were also sensitive to facial emotion. Specifically, vMORs in the theta band were significantly positive for the fearful faces, and neutral emotion was not significant from zero, indicating that deviant fearful faces induced greater theta-band oscillation compared with the standard fearful face.

A meta-analysis of the P3 amplitude showed that pictorial stimuli were more likely to induce negative biases than text stimuli (Yuan et al., 2019), implying that negative bias exists widely, not only in the automatic processing of facial emotion. Infrequently presented negative emotions can activate more intense brain activity than infrequent non-negative emotions. This may be related to the important function of the brain in detecting threats in the environment, thus increasing the chance of survival.

### Limitations and Future Directions

One limitation of our study is that we used fearful-neutral stimulus pairs as deviant-standard stimulus pairs. The conclusion of negative bias may not be convincing enough compared with the study of fearful-happy pairs, because the intensity of response to neutral emotion may be different from that to negative or positive emotion. A majority of studies involving emotion-related vMMN use negative emotion and positive emotion as stimuli. The stimuli we used had correct identification rates of only 57 and 72% for neutral and fearful emotions, respectively. Emotional faces with a higher discrimination rate might produce more reliable results in a future study.

Recently, some studies compared the differences between color change automatic processing and face automatic processing and found that the traditional vMMN and computational modeling methods reached a consistent conclusion (Stefanics et al., 2018). Future research could combine low-level visual stimuli (such as color) with high-level visual stimuli (such as facial emotion) to explore vMMN variations of different sequences.

## Summary

Our study analyzed the oddball-vMMN, reverse oddball-vMMN, and equiprobable-vMMN. We found that the vMMN effect was significant within the range of 100–350 ms from stimulus onset, but differences between the three vMMN effects were not significant. The source localization showed consistent results that the source of different sequence-vMMN had no significant difference in the whole brain. In addition, we performed time-frequency decomposition on the visual mismatch oscillations (vMORs) and found that the theta wave vMORs were significantly enhanced, but as with the vMMN effect, there were no significant differences among the three vMORs. Low-level features and visual refractoriness did not affect the vMMN/vMORs, indicating enhanced response to a rare visual stimulus rather than inhibited response to the repeated standard. Additionally, we found that the emotion-related vMMN/vMORs effect had a negative bias. These conclusions suggest that vMMN/vMORs are a good electrophysiological index to study unattended visual information processing and support the notion that vMMN is memory-trace based.

## Data Availability Statement

The original contributions presented in the study are included in the article/supplementary material, further inquiries can be directed to the corresponding author/s.

## Ethics Statement

The studies involving human participants were reviewed and approved by the institutional Research Board (IRB) of the School of Education, Guangzhou University, China. The patients/participants provided their written informed consent to participate in this study.

## Author Contributions

XZ and SF designed the experiment. XZ, YL, and YZ collected and analyzed data. XZ, LJ, YL, and YZ wrote the draft. LJ and SF contributed to the conceptualization and the editing of the manuscript. SF provided funding and resources for this research. All authors contributed to the article and approved the submitted version.

## Conflict of Interest

The authors declare that the research was conducted in the absence of any commercial or financial relationships that could be construed as a potential conflict of interest.

## Publisher’s Note

All claims expressed in this article are solely those of the authors and do not necessarily represent those of their affiliated organizations, or those of the publisher, the editors and the reviewers. Any product that may be evaluated in this article, or claim that may be made by its manufacturer, is not guaranteed or endorsed by the publisher.
